# Effect of Antioxidant Supplementation on Systemic Oxidative Status in Breeding Stallions During Intensive Semen Collection

**DOI:** 10.3390/ani16121804

**Published:** 2026-06-11

**Authors:** Chiara Del Prete, Alessio Ruggiero, Consiglia Longobardi, Monica Isabella Cutrignelli, Alessandro Vastolo

**Affiliations:** 1 Department of Human Sciences, Link Campus University, 00168 Rome, Italy; c.delprete@unilink.it; 2 Department of Veterinary Medicine and Animal Production, University of Napoli Federico II, 80137 Napoli, Italy; alessio.ruggiero@unina.it (A.R.); consiglia.longobardi@unina.it (C.L.); alessandro.vastolo@unina.it (A.V.)

**Keywords:** horse, antioxidants, volatile fatty acids, reproduction

## Abstract

Breeding stallions are subjected to increased metabolic and physiological demands during the reproductive season, which may lead to enhanced production of reactive oxygen species and disruption of redox balance. The present study evaluated the effects of dietary antioxidant supplementation on oxidative status, metabolic profile, and hindgut fermentation in stallions undergoing intensive semen collection activity. Ten adult breeding stallions were assigned either to a control group or to a treatment group receiving a commercial antioxidant supplement (Oxyliver^®^) for 60 days, followed by a 60-day post-supplementation period. Blood and fecal samples were collected at regular intervals to assess hematological, biochemical, oxidative, and fermentation parameters. Hematological and biochemical variables remained within physiological ranges throughout the study, with no significant treatment-related effects, indicating that the supplementation was safe and well-tolerated. In contrast, during the supplementation period, oxidative status was significantly influenced by the antioxidant treatment, as demonstrated by reduced lipid peroxidation and increased total antioxidant capacity (TAC) and ferric reducing antioxidant power (FRAP). These findings indicate an enhancement of systemic antioxidant defenses. In conclusion, dietary antioxidant supplementation effectively improved oxidative balance in breeding stallions without adversely affecting metabolic health, supporting its potential use as a nutritional strategy to mitigate oxidative stress associated with intensive reproductive activity.

## Simple Summary

Breeding stallions face increased physiological stress during the reproductive season, which can lead to oxidative imbalance and potentially affect their health. This study evaluated whether an antioxidant supplement could help support their physiological condition. Stallions receiving the supplement showed improved antioxidant status, with reduced markers of oxidative damage and enhanced antioxidant defenses, while no negative effects on health parameters were observed. Some changes in gut fermentation were also detected, suggesting a possible influence on intestinal microbial activity. These results indicate that antioxidant supplementation may help stallions better cope with the metabolic demands of breeding activity, representing a useful nutritional strategy in equine management.

## 1. Introduction

Forage represents the main component of the equine diet and is the primary source of nutrients for horses under most management systems, supplying fiber, energy, protein, and a substantial proportion of the daily intake of micronutrients [1,2]. Fresh forage is naturally rich in antioxidant compounds, including vitamin E, β-carotene, and other bioactive molecules that play a crucial role in maintaining systemic redox balance [3,4]. However, these compounds are highly susceptible to degradation during harvesting, drying, and storage, leading to a marked reduction in the antioxidant content of hay [3,5].

In recent years, climatic variability together with suboptimal harvesting, drying, and storage conditions has further complicated the availability of consistently high-quality hay, potentially compromising the intake of vitamins and minerals essential for metabolic homeostasis [6,7]. Although hay-based diets are generally sufficient to meet the fiber and energy requirements of horses at maintenance, they may not fully satisfy micronutrient and antioxidant requirements, particularly in animals exposed to increased physiological demands [2,8].

The nutritional requirements of horses during the breeding season are often compared to those of working horses. Animals engaged in regular physical activity or subjected to prolonged metabolic challenges exhibit increased oxygen consumption, which is associated with enhanced production of reactive oxygen species (ROS) and a greater risk of oxidative imbalance [9]. Breeding stallions represent a specific category of horses exposed to sustained physiological stress during the reproductive season, characterized by increased metabolic turnover and endocrine activity associated with regular semen collection [10]. During this period, maintaining adequate antioxidant capacity is essential to preserve systemic metabolic balance and overall health, especially when dietary antioxidant intake may be inadequate [3,4].

Dietary antioxidant supplementation has been proposed as a nutritional strategy to support redox balance by enhancing systemic antioxidant defenses and limiting oxidative damage. Although its effects have been investigated in exercising horses, limited information is currently available regarding its role in breeding stallions.

Therefore, the aim of the present study was to evaluate the effects of a commercial dietary antioxidant supplement on antioxidant status in breeding stallions during the semen collection season, through the assessment of systemic oxidative status using blood and fecal parameters.

## 2. Materials and Methods

All procedures involving animals were conducted in accordance with current regulations on animal welfare (PG/2025/001595, 6 February 2025) and were carried out as part of routine management practices at the breeding centre. No invasive or harmful procedures were performed, and all animals completed the study without adverse effects related to dietary supplementation.

### 2.1. Experimental Design

The study was conducted on ten adult breeding stallions (Salernitano, Italian heavy draft horse, and Haflinger) aged between 5 and 18 years (median age 14 years) housed at the Regional Equine Breeding Centre of Santa Maria Capua Vetere (Caserta, Italy). All animals were clinically healthy, regularly used for breeding activity, and managed under uniform housing and feeding conditions throughout the study period. Stallions were housed in individual boxes with individual drinking troughs. They had daily access to exercise paddocks and were fed hay and concentrate (Table 1). Each stallion received a concentrate ration adjusted according to body weight, while hay was provided ad libitum.

Stallions were equally divided into a control group (CTR) and a treatment group (TRT), ensuring a balanced distribution of age and breed between groups. Each group included four Salernitano stallions and one stallion of either the Haflinger or Italian Heavy Draft breed. The median age was 11.8 years in the CTR and 13.2 years in the TRT. After a 7-day adaptation period, both the control and experimental groups received the supplementation for 60 days at the same time, corresponding to one complete equine spermatogenic cycle. To evaluate the persistence of supplementation effects, the experimental protocol included an additional 60-day post-supplementation observation period without treatment.

During the supplementation phase, animals in the TRT received the commercial antioxidant supplement (Oxyliver^®,^, Candioli, Brugliaco, Italy) at a dose of 30 g/day according to the manufacturer’s instructions. Animals in the CTR received 30 g/day of maltodextrin administered as a placebo, identical in appearance and administration method (double-blind). Both Oxyliver^®^ and placebo were administered orally once daily, mixed with the concentrate ration to ensure complete intake and avoid differences in palatability or feeding behavior. The supplementation had the following composition: maltodextrin (46%), fructooligosaccharides (5%), soya (seed) protein concentrate (1%), palm oil, products and co-products of fresh fruit and vegetable processing (Melon). In addition, it contains nutritional additives per kg of product: Vitamins: Vitamin C 3a300 150,000 mg, vitamin E 3a700 75,000 IU, choline chloride 3a890 3500 mg; Amino acids: DL-Methionine, technically pure 3c301 5000 mg. Organoleptic additives: Flavourings: Bitter orange extract 2b136-ex 50,000 mg—*Curcuma longa* L.: Extract of Curcuma 2b163-ex 5330 mg—*Silybum marianum* (L.) Gaertn. = *Carduus marianus* L.: Milk thistle extract CoE 551 16,000 mg—clycine 2b17034 37,500 mg; Anti-caking agents: Colloidal silica E551b 85,000 mg; Emulsifiers: Lecithin 1c322i 34,670 mg; Stabilisers: Microcrystalline cellulose E460 10,670 mg.

**Figure 1:**
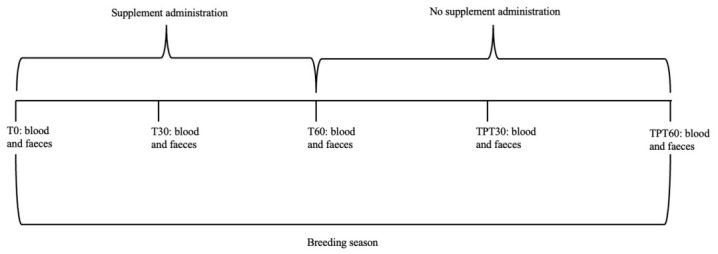
Experimental period.

The study was carried out during the breeding season, when stallions were subjected to regular semen collection (once a day), which represents a period of increased physiological and metabolic demand. Samples were collected at predefined time points during both the supplementation and post-supplementation phases. The sampling schedule included baseline evaluation at the start of supplementation (T0), followed by assessments every 30 days during supplementation (T30, T60) and time post-treatment (TPT30, TPT60) (Figure 1).

Body weight and body condition score (BCS) were recorded every fifteen days to monitor potential changes associated with supplementation. Body weight was estimated using a dedicated equine weight tape applied according to standard procedures. All measurements were performed by the same trained and qualified operator throughout the study in order to minimise inter-observer variability and reduce measurement error. BCS was determined by 2 individuals (1 constant and 1 rotating) on a scale of 1 to 5 as described by Martin-Rosset & Younge [1] (1 = very thin; 5 = obese).

### 2.2. Hematological, Biochemical Parameters and Oxidative Status Analysis

Blood samples were collected by jugular venipuncture into appropriate tubes for hematological, biochemical, and serum analyses at T0 and every 30 days throughout the supplementation and post-supplementation periods (TPT30–TPT60). At each time point, blood samples were collected in the morning before the animals carried out their routine activities and before hay administration on sampling days. The samples were kept at 4 °C and transferred to the Department of Veterinary Medicine and Animal Production, University of Napoli Federico II. The hematological examination was performed immediately with the IDEXX ProCyte Dx™ analyzer (IDEXX Laboratories, Westbrook, ME, USA) to evaluate erythrocytes (M/µL), hematocrit (%), hemoglobin (g/dL), leucocytes (K/µL), neutrophils (K/µL), lymphocytes (K/µL), monocytes (K/µL), eosinophils (K/µL), basophils (K/µL), and platelets (K/µL). To extract the serum, the tubes were centrifugated at 3000 rpm for 10 min. The serum was stored at −20 °C until analysis for biochemical evaluation, testosterone, and oxidative stress biomarkers (Total antioxidant capacity, Ferric reducing antioxidant power, and Lipid peroxidation). All the analyses were performed within 2 months. Biochemical evaluation was performed with the SAT450 clinical chemistry analyzer (KPM Analytics, Westborough, MA, USA) to evaluate urea (mg/dL), creatinine (mg/dL), glucose (mg/dL), total protein (PT; g/dL), albumin (g/dL), total bilirubin (mg/dL), aspartate aminotransfer-ase (AST; IU/L), alanine aminotransferase (ALT; IU/L), gamma-glutamyl transferase (GGT; IU/L).

Blood testosterone concentrations were measured by liquid chromatography–tandem mass spectrometry (LC-MS/MS). Briefly, blood samples were processed prior to chromatographic separation and analyzed by tandem mass spectrometry using electrospray ionization in positive mode (ESI+). Testosterone concentrations were quantified using calibration curves prepared with certified testosterone standards and expressed as ng/dL.

Total antioxidant capacity (TAC) was determined using a commercial assay kit (Abcam, ab65329, Cambridge, UK) following the manufacturer’s instructions. Briefly, serum samples were diluted 1:1000, incubated with Cu^2+^ working solution, and absorbance was measured at 532 nm using a spectrophotometer. TAC values were calculated based on a Trolox standard curve.

Ferric reducing antioxidant power (FRAP) was assessed using a commercial assay kit (Sigma-Aldrich, MAK509, Milan, Italy). Serum samples diluted 1:10 were incubated with the Fe^3+^-containing working reagent, and absorbance was measured at 610 nm after incubation at room temperature. Results were quantified according to a Fe^2+^ standard curve.

Lipid peroxidation was assessed by determining malondialdehyde (MDA) concentration according to the method of Ohkawa et al. [11]. Serum samples were initially diluted with distilled water, followed by the addition of 100 µL of 20% acetic acid, 100 µL of 0.8% thiobarbituric acid (TBA; Sigma-Aldrich, Milan, Italy), and 40 µL of 8.1% sodium dodecyl sulphate (SDS; Bio-Rad, Hercules, CA, USA). The mixtures were then incubated at 100 °C for 2 h. After centrifugation, aliquots of the supernatant were dispensed into 96-well microplates, and absorbance was measured at 532 nm using a spectrophotometer (Thermo Fisher Scientific, Rodano, Italy). MDA concentrations were quantified using a calibration curve generated with standard MDA solutions.

### 2.3. Fecal Samples Analyses

Fecal samples were collected directly from the rectum at predefined sampling times. Immediately after collection, samples were stored at −4 °C and subsequently transferred to the Laboratory of Feed and Animal Production Analysis, Department of Veterinary Medicine and Animal Productions, University of Naples Federico II. Fecal samples were collected to assess the potential impact of antioxidant supplementation on hindgut metabolic processes, including volatile fatty acid production and D- and L-lactate concentrations, which are indicative of microbial fermentation activity. After collection, samples were stored at −20 °C and processed for analysis within the time frame required to preserve fermentation characteristics. Fecal pH was measured using a calibrated pH meter after a 1:5 dilution (*w*/*v*) with calcium chloride solution (0.01 mol/L). D- and L-lactate concentrations were determined by a spectrophotometric enzymatic method at 340 nm wavelength, using a commercial Enzytec assay kit (R-Biopharm AG, Darmstadt, Germany), according to the manufacturer’s instructions.

For volatile fatty acids (VFAs) determination, the feces were centrifugated at 12,000× *g* for 10 min at 4 °C (Universal 32R, Hettich FurnTech Division DIY, Melle-Neuenkirchen, Germany), and the resulting supernatant (1 mL) was mixed with 1 mL of 0.06 mol oxalic acid. Volatile fatty acids were quantified by gas chromatography (Thermo Fisher Trace Italia SpA, Rodano, Milan, Italy) using a fused silica capillary column (Supelco, 30 m × 0.25 mm ID, 0.25 μm film thickness) and an external standard containing acetic, propionic, butyric, iso-butyric, valeric, and iso-valeric acids. The proportion of branched-chain fatty acids (BCFA) was calculated as: ((iso-butyric acid + iso-valeric acid)/total VFAs) × 100.

### 2.4. Semen Collection

Semen was collected using a pre-warmed Missouri-type artificial vagina in the presence of an estrous mare used as a teaser. Immediately after collection, semen was filtered and evaluated for volume and concentration. An aliquot of raw semen was centrifuged at 1500× *g* for 10 min to obtain seminal plasma, which was stored at −80 °C until oxidative status analyses. Semen samples diluted in commercial extender (INRA96, IMV Technologies, L’Aigle, France) were transported at 5 °C to the laboratory. The malondialdehyde (MDA) concentrations in seminal plasma were also assessed as previously mentioned.

### 2.5. Statistical Analysis

All statistical analyses were performed using R statistical software 4.6.0 (RStudio environment). Data were analyzed using linear mixed-effects models to account for repeated measures within animals. Group, time, and their interaction (group × time) were included as fixed effects, while animal was included as a random effect. When significant effects were detected, pairwise comparisons were performed using estimated marginal means with Bonferroni correction. The normality of residuals was assessed using the Shapiro–Wilk test, and residual distributions were visually inspected and considered acceptable for repeated-measures analysis. Statistical significance was set at *p* < 0.05. For consistency across tables, variability is reported as mean square error (MSE).

## 3. Results

### 3.1. Body Weight and Body Condition Score

**Figure 2:**
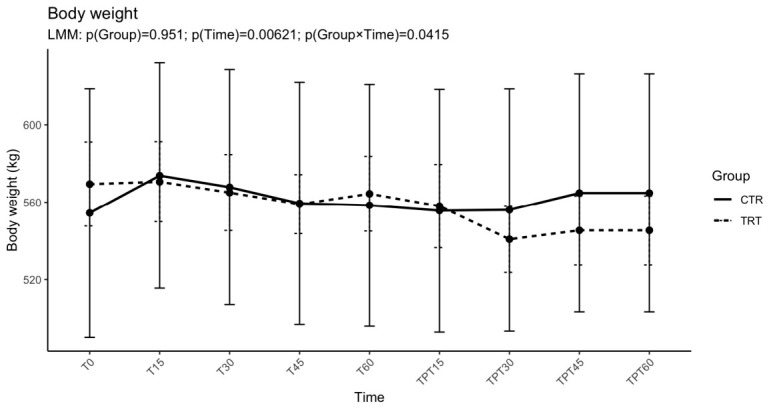
Body weight over the experimental period in control (CTR) and treatment (TRT) groups. Values are expressed as estimated marginal means. Significant effects derived from the linear mixed model are reported in the graph.

Changes in body weight are shown in Figure 2. A significant effect of time and the group × time interaction (*p* < 0.05) was observed in body weight. No differences were observed for body condition score.

### 3.2. Hematological, Biochemical Parameters and Oxidative Status

Hematological parameters are reported in Table 2. No significant effects of group or group *×* time interaction were observed. A significant effect of time was detected for lymphocytes (*p* = 0.0045), eosinophils (*p* = 0.0018), and neutrophils (*p* = 0.0233).

Biochemical parameters are reported in Table 3. A significant effect of time was detected for glucose (*p* < 0.001), total protein (*p* = 0.0013), β-globulin (*p* = 0.0250), Creatinine (*p* = 0.0002), AST (*p* = 0.0037), Albumin (*p* = 0.0021). Glucose showed a significant group × time interaction (*p* = 0.0438).

**Figure 3:**
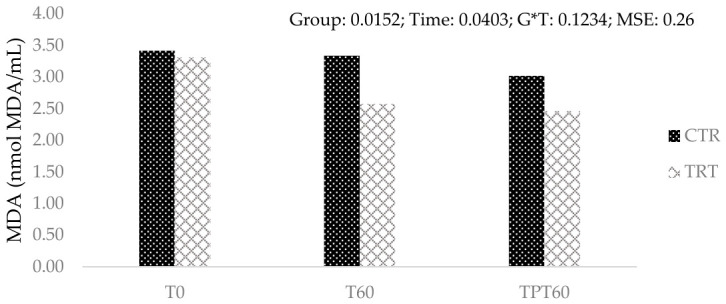
Concentrations of malondialdehyde (MDA) in the seminal plasma of stallions at different time points in the control (CTR) and treatment (TRT) groups. Values are expressed as estimated marginal means ± MSE. G*T: interaction between group and time factors.

Serum testosterone concentrations are shown in Figure 3. No significant effects of group, time, or their interaction were detected. Serum antioxidant parameters are reported in Table 4. Serum oxidative parameters are reported in Table 4. MDA concentrations were significantly affected by group (*p* = 0.0012), while TAC showed significant effects of group (*p* = 0.0416), time (*p* = 0.0104), and group × time interaction (*p* = 0.0310). FRAP was significantly influenced by both group (*p* = 0.0373) and time (*p* = 0.0246).

Seminal plasma MDA concentrations are shown in Figure 3. Significant effects of group (*p* = 0.0152) and time (*p* = 0.0403) were detected. The TRT showed lower MDA concentrations than the CTR at T60, and this difference was maintained at TPT60.

### 3.3. Fecal Samples End-Prodocts

Fecal fermentation parameters are reported in Table 5. Volatile fatty acids (VFAs) were significantly affected by time. Acetate and propionate concentrations decreased over time (*p* = 0.0001 and *p* = 0.0039, respectively). Total VFA concentrations also decreased during the post-treatment period (*p* = 0.0001). Iso-butyrate and iso-valerate showed time-dependent variations (*p* < 0.0096 and *p* < 0.0145), while valerate decreased over time (*p* = 0.0008). A significant (*p* = 0.0082) group effect was observed for butyrate.

D-lactate concentrations showed a significant (*p* < 0.001) increase over time, whereas L-lactate was not significantly affected.

## 4. Discussion

The present study investigated the effects of dietary antioxidant supplementation on metabolic profile, oxidative status, and fecal parameters in breeding stallions during the reproductive season. Although the number of animals included was limited due to the restricted availability of breeding stallions managed under homogeneous conditions during the breeding period, the findings provide preliminary evidence supporting the effects of antioxidant supplementation on systemic oxidative status.

A significant effect of time and a significant group x time interaction were observed for body weight, whereas BCS remained relatively stable throughout the study period. The significant interaction observed for body weight indicates differences in temporal trends between groups; however, these variations were not accompanied by corresponding changes in body condition score. Previous studies [12,13] have shown that body weight in horses may fluctuate over time because of metabolic and management-related factors without necessarily reflecting changes in fat deposition or nutritional status. In particular, BCS has been recognized as a more reliable indicator of body fat reserves than body weight alone, since variations in body weight are not always associated with changes in body condition. In this study, the stability of BCS suggests that the observed differences in body weight were not associated with meaningful alterations in body condition, supporting the interpretation that the group × time interaction did not reflect a substantial effect of supplementation on body condition.

Hematological variables remained largely within normal physiological ranges [14]. The absence of consistent group and interaction effects suggests that supplementation did not induce inflammatory or hematological disturbances. A significant effect of time was detected for lymphocytes, eosinophils, and neutrophils, indicating temporal variation in specific leukocyte populations. Similar findings have been reported in horses, where leukocyte subsets are known to fluctuate over time in response to exercise, stress, and management conditions [15,16]. These variations are generally considered part of normal immune adaptation rather than evidence of pathological processes.

Biochemical parameters in horses are known to fluctuate over time under normal conditions. In particular, variations in total protein and albumin have been associated with fluid shifts and metabolic adjustments related to feeding, management, and sampling conditions [16].

Temporal changes in glucose concentration are commonly reported in horses and may reflect normal fluctuations influenced by handling, sampling-related stress, and metabolic adaptation over time [16,17]. The significant interaction observed for glucose suggests differences in temporal patterns between groups; however, considering the intrinsic variability of this parameter, these changes are more likely related to adaptive responses rather than to a consistent effect of supplementation [17].

AST concentrations may increase in response to training, metabolic adaptation, or subclinical conditions, although moderate fluctuations are frequently observed even in clinically healthy horses [16]. In the present study, the absence of group effects and the maintenance of values within physiological ranges suggest that AST variation was more likely related to normal biological variability than to a specific hepatic response to supplementation. Creatinine concentrations, generally considered stable indicators of renal function, may also show limited variation related to metabolic status and individual variability without necessarily indicating renal impairment [18]. Likewise, albumin, a marker of hepatic synthetic function, showed temporal variation while remaining within physiological limits, supporting the absence of clinically relevant hepatic dysfunction.

The observed changes in total protein and β-globulins may be partially associated with temporal modulation of the immune system. This interpretation is supported by the significant time effect observed for leukocyte populations, including lymphocytes, eosinophils, and neutrophils. In horses, leukocyte dynamics are strongly influenced by endocrine responses, particularly cortisol, which can induce neutrophilia and lymphopenia under conditions of stress or moderate exercise [16,19]. It is important to note that most available studies describing hematological and biochemical variations in horses refer to racehorses or athletic horses, in which exercise and training are major influencing factors [16]. In contrast, data specifically related to breeding stallions are still limited. Therefore, the temporal variations observed in the present study may reflect adaptive changes associated with management conditions and reproductive activity rather than exercise-induced effects.

Oxidative stress in horses is defined as an imbalance between reactive oxygen species (ROS) production and antioxidant defenses, potentially leading to damage to lipids, proteins, and DNA [20]. In this context, malondialdehyde (MDA) is widely recognized as a marker of lipid peroxidation and oxidative damage to cellular membranes [11], whereas total antioxidant capacity (TAC) and ferric reducing antioxidant power (FRAP) reflect the overall antioxidant potential of the organism. Previous studies [20,21] have demonstrated that physiological challenges, such as exercise or metabolic stress, may increase oxidative stress and induce adaptive changes in antioxidant systems. In particular, increases in lipid peroxidation and modulation of antioxidant defenses have been observed under conditions of enhanced ROS production. Antioxidants such as vitamin E and vitamin C play a central role in maintaining redox balance. Vitamin E acts primarily as a lipid-soluble antioxidant, protecting cell membranes from oxidative damage, whereas vitamin C contributes to the regeneration of oxidized vitamin E and supports the antioxidant network [21,22]. The combined presence of these antioxidants may enhance the capacity to neutralize ROS and limit lipid peroxidation. In this context, the tested supplement (Oxyliver^®^, Candioli, Beinasco, Italy) contains both vitamin E and vitamin C, potentially contributing to the modulation of oxidative status.

Lower MDA concentrations observed in the supplemented group, together with changes in TAC and FRAP, may indicate modulation of systemic redox balance during the supplementation period. Overall, the observed patterns are consistent with adaptive modulation of oxidative status over time.

Volatile fatty acids (VFAs), including acetate, propionate, and butyrate, are the main end-products of microbial fermentation in the equine hindgut and contribute to intestinal homeostasis and energy metabolism [23]. In this study, several fermentation parameters showed significant temporal variations, suggesting that hindgut microbial activity changed throughout the experimental period independently of treatment effects. In particular, acetate, propionate, valerate, and total SCFA concentrations were significantly affected by time, indicating a general modulation of fermentative activity during the study period. Among VFAs, butyrate was the only parameter showing a significant group effect, with lower concentrations observed in the TRT throughout the experimental period. However, because lower butyrate values were already present at baseline (T0) and no significant group x time interaction was detected, this finding should be interpreted cautiously, as it may reflect pre-existing differences between groups rather than a direct effect of supplementation. Nevertheless, butyrate remains an important metabolite involved in intestinal epithelial function and microbial metabolic homeostasis [24,25,26,27,28]. Similarly, D-lactate showed a significant time effect, whereas no significant effects of group or group x time interaction were detected, suggesting temporal variation during the experimental period. Despite the presence of fructooligosaccharides (5%) in the supplement, which have previously been reported to modulate hindgut microbial activity and influence volatile fatty acid production in horses [29], no consistent differences between groups were observed for the main fermentation end-products. Therefore, under the conditions of the present study, supplementation did not appear to substantially affect hindgut fermentation patterns.

## 5. Conclusions

In conclusion, antioxidant supplementation with Oxyliver^®^ in breeding stallions under intensive reproductive activity was well tolerated and did not negatively affect hematological or biochemical parameters. The main findings of the present study concern the modulation of oxidative status, with variations observed in MDA, FRAP, and TAC during the supplementation period, indicating an effect of the product on redox balance. These changes are consistent with the antioxidant properties of the supplement, which contains vitamin E and vitamin C, known to act synergistically in limiting oxidative damage and supporting the antioxidant network.

Overall, the results suggest that antioxidant supplementation may contribute to the regulation of oxidative processes in breeding stallions subjected to physiological stress conditions, without compromising their general health status.

## Figures and Tables

**Table 1:** Composition of the mixed hay and concentrate (% as fed).

Item	Mixed Hay	Concentrate
DM	91.5	87.0
CP	6.73	13.5
CF	31.3	6.80
NDF	57.8	42.5
ADF	39.2	23.3
ADL	12.2	5.1
EE	1.34	3.98
Ash	9.20	5.98

DM: dry matter; CP: crude protein; CF: crude fiber; NDF: neutral detergent fiber; ADF: acid detergent fiber; ADL: acid detergent lignin. Ingredient: Mixed hay (Grass hay (ryegrass, fescue): 80–90%; Alfalfa hay: 10–20%); Concentrate: Durum wheat bran, wheat middlings, flaked corn, oats, barley flour, flaked barley, soybean meal, calcium carbonate, cane molasses, flaked faba beans, carob seeds, soy vegetable oils and fats, sodium chloride (salt).

**Table 2:** Hematological parameters in breeding stallions.

	Red Blood Cells (10^6^/µL)		Hemoglobin (g/dL)	
	CTR	TRT	CTR	TRT
T0	8.36	8.26	15.3	15.3
T30	7.44	8.06	12.3	14.6
T60	7.67	7.98	14.0	14.5
TPT30	7.70	8.38	13.8	15.4
TPT60	7.76	8.31	13.6	14.8
Group	0.7430		0.7495	
Time	0.1148		0.0530	
G*T	0.4418		0.5454	
MSE	0.56		1.25	
	Hematocrit (%)		Platelets (10^3^/µL)	
	CTR	TRT	CTR	TRT
T0	40.1	39.8	145	160
T30	35.3	38.3	148	145
T60	37.1	37.8	125	144
TPT30	37.1	39.9	132	146
TPT60	36.8	39.2	137	144
Group	0.7865		0.3923	
Time	0.1024		0.1492	
G*T	0.5842		0.6427	
MSE	3.07		15.2	
	White Blood Cells (10^3^/µL)		Neutrophils (10^3^/µL)	
	CTR	TRT	CTR	TRT
T0	7.82	6.15	4.65	3.77
T30	7.01	6.17	4.17	3.95
T60	7.12	6.72	4.11	4.46
TPT30	6.35	6.67	3.56	4.25
TPT60	7.50	7.71	4.69	5.39
Group	0.4722		0.8535	
Time	0.0907		0.0233	
G*T	0.2148		0.2797	
MSE	0.92		0.80	
	Lymphocytes (10^3^/µL)		Monocytes (10^3^/µL)	
	CTR	TRT	CTR	TRT
T0	2.69	2.04	0.43	0.46
T30	2.38	1.83	0.37	0.32
T60	2.55	1.87	0.36	0.30
TPT30	2.35	1.97	0.33	0.36
TPT60	2.29	1.69	0.33	0.35
Group	0.1558		0.0755	
Time	0.0045		0.9683	
G*T	0.5458		0.592	
MSE	0.19		0.08	
	Eosinophils (10^3^/µL)		Basophils (10^3^/µL)	
	CTR	TRT	CTR	TRT
T0	0.05	0.04	0.01	0.02
T30	0.07	0.07	0.01	0.01
T60	0.06	0.08	0.02	0.01
TPT30	0.08	0.10	0.02	0.01
TPT60	0.16	0.23	0.03	0.03
Group	0.8717		0.5412	
Time	0.0018		0.0028	
G*T	0.3505		0.7488	
MSE	0.08		0.01	

CTR: Control group; TRT: treatment group; G*T: interaction between group and time factors. MSE: mean square error.

**Table 3:** Biochemical parameters in breeding stallions.

	Urea (mg/dL)		Creatinine (mg/dL)	
	CTR	TRT	CTR	TRT
T0	31.3	29.0	1.33	1.13
T30	28.8	23.0	1.29	1.03
T60	32.2	25.5	1.27	1.18
TPT30	30.2	21.7	1.23	1.13
TPT60	29.4	27.5	1.33	1.30
Group	0.1706		0.0876	
Time	0.4259		0.0002	
G*T	0.8099		0.4112	
MSE	4.96		0.09	
	Glucose (mg/dL)		AST (IU/L)	
	CTR	TRT	CTR	TRT
T0	98.4	92.5	263	317
T30	106	96.7	257	279
T60	110	101	311	290
TPT30	80.6	75.7	250	246
TPT60	96.0	92.7	229	240
Group	0.7485		0.7505	
Time	<0.001		0.0037	
G*T	0.0438		0.6168	
MSE	8.02		34.4	
	ALT (IU/L)		GGT (IU/L)	
	CTR	TRT	CTR	TRT
T0	6.80	9.25	9.46	9.80
T30	7.80	7.75	9.52	9.05
T60	10.8	7.75	10.2	10.7
TPT30	5.80	7.25	9.34	10.6
TPT60	7.40	8.50	9.66	15.1
Group	0.7469		0.6353	
Time	0.1556		0.2109	
G*T	0.1243		0.2454	
MSE	2.13		2.69	
	Total Protein (g/dL)		Albumin (g/dL)	
	CTR	TRT	CTR	TRT
T0	5.99	6.38	3.13	3.18
T30	5.59	5.76	2.87	2.76
T60	6.31	5.94	3.25	3.07
TPT30	5.72	5.92	2.99	3.07
TPT60	6.21	6.17	2.95	3.05
Group	0.4504		0.8452	
Time	0.0013		0.0021	
G*T	0.1548		0.2863	
MSE	0.34		0.22	
	Alfa 1 (g/dL)		Alfa 2 (g/dL)	
	CTR	TRT	CTR	TRT
T0	0.15	0.24	0.74	0.75
T30	0.16	0.20	0.67	0.75
T60	0.19	0.18	0.74	0.65
TPT30	0.17	0.22	0.64	0.66
TPT60	0.20	0.18	0.73	0.69
Group	0.7671		0.7444	
Time	0.4496		0.5211	
G*T	0.8989		0.6103	
MSE	0.03		0.11	
	Beta (g/dL)		Gamma (g/dL)	
	CTR	TRT	CTR	TRT
T0	1.13	1.12	0.82	0.82
T30	1.00	1.10	0.91	1.00
T60	1.19	1.15	0.99	0.91
TPT30	1.05	1.12	0.89	0.92
TPT60	1.33	1.17	1.04	1.10
Group	0.2377		0.5688	
Time	0.0250		0.4712	
G*T	0.6730		0.9087	
MSE	0.33		0.56	
	Albumin/Globulin Ratio			
	CTR	TRT		
T0	1.12	1.11		
T30	1.11	0.93		
T60	1.07	1.08		
TPT30	1.10	1.08		
TPT60	0.92	1.00		
Group	0.2237			
Time	0.9503			
G*T	0.8894			
MSE	0.43			

CTR: Control group; TRT: treatment group; G*T: interaction between group and time factors. MSE: mean square error.

**Table 4:** Testosterone and antioxidant parameters in serum of control and treatment groups.

	Testosterone (ng/dL)		TAC (nmol Trolox/well)	
	CTR	TRT	CTR	TRT
T0	30.9	30.9	268	272
T30	42.4	47.6	270	334
T60	50.3	52.4	269	366
TPT30	47.5	44.3	282	325
TPT60	50.9	49.6	270	292
Group	0.5352		0.0416	
Time	0.0673		0.0104	
G*T	0.2915		0.0310	
MSE	14.76		30.22	
	FRAP (µmol Fe^2+^ equivalents/L)		MDA (nmol MDA/mL)	
	CTR	TRT	CTR	TRT
T0	355	349	4.78	4.94
T30	348	448	4.59	3.32
T60	378	444	4.34	3.42
TPT30	387	398	4.43	3.92
TPT60	375	384	4.51	4.30
Group	0.0373		0.0012	
Time	0.0246		0.0835	
G*T	0.7826		0.6918	
MSE	21.15		0.36	

CTR: Control group; TRT: treatment group; G*T: interaction between group and time factors. TAC: Total antioxidant capacity; FRAP: Ferric Reducing Antioxidant Power; MDA: malondialdehyde; MSE: mean square error.

**Table 5:** Final fermentation production of control and treatment groups.

	Acetate		Propionate	
	CTR	TRT	CTR	TRT
T0	25.5	30.7	8.02	8.26
T30	25.5	29.7	7.63	7.97
T60	31.5	28.3	9.09	7.06
TPT30	22.9	21.6	7.36	6.30
TPT60	20.5	20.4	6.55	5.74
Group	0.5352		0.4436	
Time	0.0001		0.0039	
G*T	0.1914		0.2211	
MSE	4.32		1.19	
	Iso-Butyrate		Butyrate	
	CTR	TRT	CTR	TRT
T0	1.15	0.95	3.97	2.01
T30	0.83	0.79	2.74	2.10
T60	1.14	0.90	3.32	1.88
TPT30	0.80	0.63	2.71	1.57
TPT60	0.65	0.69	1.97	1.44
Group	0.4983		0.0082	
Time	0.0096		0.0785	
G*T	0.7826		0.4924	
MSE	0.26		0.96	
	Iso-Valerate		Valerate	
	CTR	TRT	CTR	TRT
T0	1.25	0.92	0.58	0.57
T30	1.22	0.91	0.54	0.51
T60	1.44	1.04	0.65	0.48
TPT30	1.26	0.60	0.61	0.35
TPT60	1.03	0.56	0.39	0.29
Group	0.1349		0.4178	
Time	0.0145		0.0008	
G*T	0.5906		0.1275	
MSE	1.02		0.11	
	BCFA		SCFA	
	CTR	TRT	CTR	TRT
T0	5.94	4.29	40.5	43.4
T30	5.25	4.05	38.5	41.2
T60	5.30	4.93	47.2	39.6
TPT30	5.37	3.91	35.7	31.0
TPT60	5.16	4.29	30.9	29.2
Group	0.2581		0.5496	
Time	0.6855		0.0001	
G*T	0.6511		0.2688	
MSE	0.98		6.14	
	D-Lactate		L-Lactate	
	CTR	TRT	CTR	TRT
T0	0.92	0.67	5.03	5.72
T30	5.49	4.75	4.41	3.91
T60	5.02	4.55	3.57	2.96
TPT30	4.21	4.03	2.18	3.50
TPT60	1.81	1.69	3.40	2.69
Group	0.3779		0.7922	
Time	<0.001		0.2085	
G*T	0.9182		0.2067	
MSE	1.03		1.18	
	pH			
	CTR	TRT		
T0	6.34	6.39		
T30	6.49	6.89		
T60	6.50	6.78		
TPT30	6.26	6.58		
TPT60	6.54	6.59		
Group	0.1589			
Time	0.0685			
G*T	0.4009			
MSE	0.27			

CTR: control group, TRT: treatment group. BCFA: branched chain fatty acids; SCFA: short chain fatty acids. G*T: interaction between group and time factors. MSE: mean square error.

## Data Availability

Data is provided by the corresponding author.

## Abbreviations

The following abbreviations are used in this manuscript:

TAC	Total antioxidant capacity
FRAP	Ferric reducing antioxidant power
ROS	Reactive oxygen species
CTR	Control group
TRT	Treatment group
BCS	Body condition score
DM	Dry matter
CP	Crude protein
CF	Crude fiber
NDF	Neutral detergent fiber
ADF	Acid detergent fiber
ADL	Acid detergent lignin
PT	Total protein
AST	Aspartate aminotransferase
ALT	Alanine aminotransferase
GGT	Gamma-glutamyl transferase
MDA	Malondialdehyde
TBARSs	Thiobarbituric acid reactive substances
VFAs	Volatile fatty acids
BCFAs	Branched-chain fatty acids
SCFAs	Short-chain fatty acids
